# Induced resistance to Fusarium wilt of banana caused by Tropical Race 4 in Cavendish cv Grand Naine bananas after challenging with avirulent *Fusarium* spp.

**DOI:** 10.1371/journal.pone.0273335

**Published:** 2022-09-21

**Authors:** Fernando A. García-Bastidas, Rafael Arango-Isaza, Hector A. Rodriguez-Cabal, Michael F. Seidl, Giulio Cappadona, Rafael Segura, Maricar Salacinas, Gert H. J. Kema

**Affiliations:** 1 Laboratory of Plant Breeding, Wageningen University, Wageningen, The Netherlands; 2 Escuela de Biociencias, Faculta de Ciencias, Universidad Nacional de Colombia- Sede Medellín (UNALMED), Medellín, Colombia; 3 Corporación para Investigaciones Biológicas, unidad de biotecnología Vegetal (CIB), Medellín, Colombia; 4 Facultad de Ciencias Exactas y Naturales, Grupo Agrobiotecnologia. Universidad de Antioquia, Medellin, Colombia; 5 Laboratory of Phytopathology, Wageningen University, Wageningen, The Netherlands; 6 Soil Geography and Landscape, Wageningen University, Wageningen, The Netherlands; Benemérita Universidad Autónoma de Puebla: Benemerita Universidad Autonoma de Puebla, MEXICO

## Abstract

In the last century, Fusarium wilt of banana (FWB) destroyed the banana cultivar Gros Michel. The Cavendish cultivars saved the global banana industry, and currently they dominate global production (~50%) and the export trade (~95%). However, a new strain called Tropical Race 4 (TR4) surfaced in the late 1960’s, spread globally and greatly damages Cavendish plantations as well as manifold local varieties that are primarily grown by small holders. Presently, there is no commercially available replacement for Cavendish and hence control strategies must be developed and implemented to manage FWB. Here, we studied whether it is possible to induce resistance to TR4 by pre-inoculations with different *Fusarium* spp. Only pre-treatments with an avirulent Race 1 strain significantly reduced disease development of TR4 in a Cavendish genotype and this effect was stable at various nutritional and pH conditions. We then used transcriptome analysis to study the molecular basis of this response. Several genes involved in plant defence responses were up-regulated during the initial stages of individual infections with TR4 and Race 1, as well as in combined treatments. In addition, a number of genes in the ethylene and jasmonate response pathways as well as several gibberellin synthesis associated genes were induced. We observed upregulation of RGA2 like genes in all treatments. Hence, RGA2 could be a key factor involved in both R1 and TR4 resistance. The data support the hypothesis that activating resistance to Race 1 in Cavendish bananas affects TR4 development and provide a first insight of gene expression during the interaction between various *Fusarium* spp. and banana.

## Introduction

Banana (*Musa* spp.) is the fourth most important crop after rice, wheat, and corn [1, 2]. Most cultivated bananas are derived from inter and intra specific hybridizations between two wild seeded diploid ancestors, *Musa acuminata* (AA, 2n = 22) and *M*. *balbisiana* (BB, 2n = 22) [1, 3] such as plantains (AAB) and cooking bananas (ABB). Bananas and plantains are a major staple food crop in many producing countries with an annual production of approximately 120 million tons in 2020 [2]. The popular dessert bananas such as ‘Gros Michel’ (from the 18e century to mid-1950’s) and ‘Cavendish’ clones are sterile triploids (AAA). The latter dominates global production (~50%) and the export trade (~95%) [4]. This is due to the resistance of Cavendish varieties to the race 1 of Fusarium wilt of banana (FWB) that wiped-out ‘Gros Michel’ plantations in Central and Latin America [5, 6] in the previous century [7]. However, FWB is once again threatening the banana industry because Cavendish bananas and manifold local varieties are susceptible to a strain of *Fusarium* colloquially called Tropical Race 4 (TR4). Previously, FWB was considered to be caused by genotypes of *F*. *oxysporum* f.sp. *cubense* (*Foc*), referred as Races. However, recent taxonomical revisions have concluded that, as expected from the polyphyletic nature of *Foc* [8–11], these actually represent a suite of *Fusarium* species and thus, TR4 has been renamed as *F*. *odoratissimum* [12]. This newly renamed species is indigenous across Indonesia and is disseminating around the world [12–18].

The nomenclature of the recognized races is based on specific interactions incurred by these *Fusarium* spp. on a small set of banana varieties [19–21]. Race 1 (R1), pathogenic to banana genotypes ‘Gros Michel’ and ‘silk’ among others, has different genetic origins and was responsible for the aforementioned devastating epidemic that caused the collapse of the ‘Gros Michel’ based banana industry in the 1950’s [22]. Race 2 (R2) infects banana genotypes of the Bluggoe group and other members with an ABB genome [18, 20, 23–27]. Race 4, however, affects the R1 resistant cultivars from the Cavendish group as well as other banana genotypes susceptible to R1 and R2. Race 4 strains are currently divided into subtropical race 4 (STR4) that affects Cavendish under specific abiotic constraints such as water logging [18, 20, 23, 24, 26, 28–31] and TR4.

The collapse of Cavendish plantations [7, 32] calls for new resistant varieties and sustainable disease management strategies. However, little is known about sources of resistance to TR4, and consequently commercially acceptable resistant banana varieties are currently unavailable. Garcia-Bastidas et al. (2019) [33] have surveyed resistance in hundreds of banana accessions under greenhouse conditions and concluded that less than 20% showed the required level of resistance to TR4. However, the genetic make-up of these accessions is unknown and the first resistance genes to FWB were only recently discovered and deployed [34]. This situation urges for exploration of alternative strategies to rapidly reduce the impact of TR4.

Induced systemic resistance (ISR) and systemically acquired resistance (SAR) are common responses of plants to certain non-pathogenic strains of microorganisms like bacteria, fungi, and viruses as well as host–incompatible nematodes [35]. The resistance can be localized, or systemic in which case the entire plant is less susceptible to later pathogen infections [36]. ISR and SAR trigger several plant defence mechanisms like production of antimicrobial compounds, formation of reactive oxygen species (ROS), and synthesis of pathogenesis-related (PR) proteins [37]. Naturally induced SAR and ISR have been observed in several plant species, however, it has been reported more frequently in dicots than in monocot plants [35, 38–48]. Few studies have been reported on banana describing ISR against nematodes [49], banana bunchy top virus [50–52], and *Fusarium* strains [53]. Wu et al. (2013) [54] showed the effect of a non-pathogenic strain as activator of SAR on Cavendish in *in vitro* plants. Thus, knowledge of ISR or SAR induced disease protection in banana is very limited, particularly in the Banana-*Fusarium* pathosystem.

We explored the potential of SAR and ISR by treating Cavendish banana plants with an avirulent R1 *Fusarium* strain as well as six *Fusarium* spp.–including four that are pathogenic on other crops but not on banana—to prevent or slow down pathogenesis of TR4. Additionally, we performed a transcriptome analysis to study gene regulation during the initial stages of infection upon inoculations with individual TR4 and R1 strains as well as various challenge inoculation with both strains and also determined the effects of abiotic stresses on cross protection.

## Materials and methods

### Plant material

Tissue cultured Cavendish ‘Grand Naine’ plants were obtained from Rahan Meristems Ltd. (Western Galilee, Israel), and then acclimatized by growing until they were 30 cm in height and had 5–6 true leaves (2.5 months). Controlled greenhouse conditions comprised 2 weeks at 28±2°C, 16h light, and ~85% relativity humidity (RH) for acclimatization and 8 weeks in a greenhouse at 26 ±2°C and ~80% relativity humidity under 16h light for hardening the plants. Henceforward, they were transplanted to new 2 L pots in sterilised sand and placed in the same greenhouse at 85% RH for 3 months until phenotyping.

### Fungal isolates

Nine *Fusarium* strains were used from the Wageningen University & Research collection (Table 1), including a R1 strain from Brazil that is used for routine phenotyping in the Embrapa breeding program in Cruz das Almas. This strain was isolated from a *Musa* spp. group ABB-‘Maça’, and according to the latest information has an unknown genotype [8, 12] as it differs from all earlier described vegetative compatibility groups [29, 55–58]. In addition we used a R2 strain originating from *a* Musa spp. group ABB-Bluggoe currently known as *F*. *tardichlamydosporum* [12] and the reference *F*. *odoratissimum* II5, TR4 strain (http://www.broadinstitute.org/). Finally, we used four *Fusarium oxysporum* formae speciales that are pathogenic on melon, onion, tomato and gladiolus but are non-pathogenic on banana as well as two well-known biocontrol strains (Table 1). To obtain fresh mycelium, isolates were grown at 25 ± 2°C on potato dextrose agar (PDA) for 4–5 days in the dark and then 5 mm plugs were taken from the edge of a colony to inoculate liquid Mung bean media in order to obtain conidia for inoculation [33]. Final inoculum was prepared after filtration through cheesecloth after 4–5 days to remove mycelial fragments and adjusted to 10^6^ conidia.mL^-1^.

**Table 1:** Origins and characteristics of the *Fusarium* spp. strains used in this study.

*Fusarium* spp.^1^	Code	Host	VCG^2^	Origin	Provider
FOSC clade 4	R1	Banana	na	Cruz das Almas, Bahía, (Brasil)	M. Dita, Netherlands
*F*. *tardichlamydosporum*	R2	Banana	0124	USA	K. O’Donnell, USA
*F*. *odoratissimum*	TR4	Banana	01213	Indonesia	R. Ploetz, USA
*Fo*. *melongenae*	*Fom*	Eggplant	n.a.	Israel	Unpublished
*Fo*. *lycopersici*	*Fol*	Tomato	n.a.	Netherlands	Unpublished
*Fo*. *cepae*	*Foce*	Onion	n.a.	Australia	Unpublished
*Fo*. *gladioli*	*Fog*	Gladiola	n.a.	-	Unpublished
FOSC Clade 3	*Fo47*	Biocontrol	n.a.	France	Lemanceau et al., 1991 [110]
-	*Fo618-12*	Biocontrol	n.a.	Netherlands	Postma and Luttikholt, (1996) [111]

^1^Names according to the latest classification by Maryani *et al*., (2018).

^2^ VCG = vegetative compatibility group.

### Co-inoculations of banana plants

Challenge experiments were carried out with non-pathogenic or incompatible strains as a putative resistance inducer and TR4 as the challenge strain (Table 2). Banana plants were uprooted, cleaned by rinsing the roots with water and immersed into the inducer inoculum for 30 min followed by immersion in the challenge TR4 spore suspension for 30 min. Thereafter, the plants were potted into sterile sand in 2L pots. Controls included: (i) plants that were only inoculated with TR4, or (ii) with the non-pathogenic strains, or (iii) exposed to a reverse experiment where TR4 inoculation was followed by R1 or non-pathogenic *Fusarium* strains as challengers. To measure the duration of induced resistance by R1 the challenge inoculation with TR4 was conducted at 30 min, 3h and 1, 2, 5 and 10 days after R1 inoculation. Controls included the same number of plants inoculated with TR4 and mocks, treated with water.

**Table 2:** Disease Indexes of banana plants treated with different pathogenic and non-pathogenic strains of *Fusarium spp*.

Treatments	Mock^1^	TR4	R1	R2	*Fom*	*Fol*	*Foce*	*Fog*	*Fo47*	*Fo618*
*F*.spp.^2^	0	100	5	60	0	20	0	0	0	0
*F*.spp + TR4^3^	-	-	20	75	60	60	50	50	95	80
TR4 + R1^4^	-	-	95	-	-	-	-	-	-	-

^1^water control. TR4: Tropical Race 4, R1: Race 1, R2: Race 2, Fom: *Fusarium oxysporum* f.sp. *melongenae*, Fol: *Fusarium oxysporum* f.sp. *lycopersici*, Foce: *Fusarium oxysporum* f.sp. *cepae*, Fog: *Fusarium oxysporum* f.sp. *gladioli*.

^2^Disease index of inoculated plants with an individual strain, calculated as described in Materials and Methods,

^3^Disease index of co-inoculated plants first with the nonpathogenicstrain and then with TR4,

^4^Disease index of co-inoculated plants first with the pathogenic TR4 and then with the R1.

### Co-inoculations under different abiotic conditions

We also tested whether induced resistance upon inoculations with R1 was maintained under different abiotic conditions. Therefore, after inoculations as described above, plants were transferred to a light textured (2% clay) and medium fertile (soil organic matter 2.6%; pH 5.2; Cation Exchange Capacity 16mmol g^-1^) soil with either pH 6.0 or 5.2 and were supplemented with three levels of nitrogen, obtained through weekly fertilizations with ammonium nitrate (NH_4_NO_3_) at 0, 0.28 and 0.85 g/plant/week. The pH levels were generated by applying calcium carbonate (CaCO_3_) amendments.

### Disease assessment

Plants were monitored weekly for FWB development, and a final evaluation was performed at 6 weeks after infection (wai), when progression of wilting stopped in the TR4 inoculated controls. Final scoring included external and internal evaluation of the disease progress [33], comprising foliar chlorosis, pseudostem splitting and deformation of new leaves. Internally, the infected area of the corm was photographed and quantified by ImageJ analyses and disease indexes (DIs) were calculated. These scores have been incorporated to a disease index (DI) according to McKinney’s formula: Ʃ(score in the scale * frequency)/(total number of plants * maximum class in the scale)] * 100. Note, the minimum class of the scale has to be 0 [33].

### Statistical lay-out

All experiments were arranged in a completely randomized block design with at least two replicates. Data were subjected to analysis of variance and least significant differences (LSD, p = 0.05) were calculated to determine the effects of the treatments. The experiments involving the initial inoculations of *Fusarium* spp. and the R1 + TR4 interactions were done in three plants with two replicates. The experiment involving abiotic factors was performed in a factorial design with four replicates and individual pots as experimental units (S1 Table). Collected data included pseudostem diameter (Dia), Foliage (FA), dry weight (DW) and plant height (He). Pseudostem diameter was selected as the best plant growth variable for data analyses.

### Transcriptome analysis of the interaction between R1 and TR4 on ‘Grand Naine’

For transcriptome analysis the challenge TR4 strain was inoculated after 30 min of Race 1 pre-inoculation. Then, complete rhizome (Corms and roots) samples of the inoculated plants and controls were taken for RNA isolation at 0 h, 3 h, 1 days, and 2 days post TR4 inoculation. Rhizomes tissues were frozen in liquid nitrogen and maintained at -80°C in a conventional freezer until all the sampling was complete.

Total RNA was isolated by grinding 100 g of rhizome with pestle and mortar in liquid nitrogen, and using the RNeasy^®^, Qiagen (Chatsworth, CA) kit, following the manufacturer’s instructions. RNA concentration was measured with the Kit Quant-iT^®^ RiboGreen^®^ (Invitrogen, Karlsruhe, Germany) in a Tecan^®^ infinite M200 microplate reader (Salzburg, Austria) (ex. 490nm, em. 530nm). As in vitro controls, RNA was isolated from pure colonies of TR4 and R1 taken from PDA plates. Samples were used for library preparation with Illumina TruSeq^®^ stranded mRNA, followed by sequencing on a HiSeq 2500 machine (125 bp paired-end) at the cluster of Applied Bioinformatics-Bioscience at WUR. RNAseq sequences were submitted to The European Nucleotide Archive (ENA) project No.PRJEB55104.

### Transcriptome assembly and analysis

We compared the transcript levels between different treatments at time 0 h, 3 h, 1 days, and 2 days post inoculation using the mock inoculation treatment as control. RNASeq read abundances per sample were summarized for banana transcripts based on the V2.0 annotation, using Kallisto v0.42.4, [59]. Transcriptome analysis was performed using R software [60]. Principal component analysis was performed using regularized Log transformed TPM (Transcripts Per Kilobase Million) values as implemented in the DESeq2 package [61] and differential gene expression between individual conditions was analysed using the same package. Only genes that had a log-Fold Changes (*LogFC*) of greater or equal to 2 for up-regulated and less or equal to -2 for down-regulated and adjusted p-value of 0.01 (padj) were considered.

Genes were grouped in four main clusters according to their functional annotations retrieved from the Gene Ontology and KEGGs databases. Hierarchical clustering analysis of the differential gene expression was performed using MultiExperiment Viewer version 4.8.1 (https://mev.tm4.org/#/about), based on log Fold Changes (LogFC) values(Pearson Correlation, average linkage clustering [62]).

## Results

### The incompatible interaction between Cavendish ‘Grand Naine’ and Fusarium R1 incurs induced resistance to TR4

**Fig 1:**
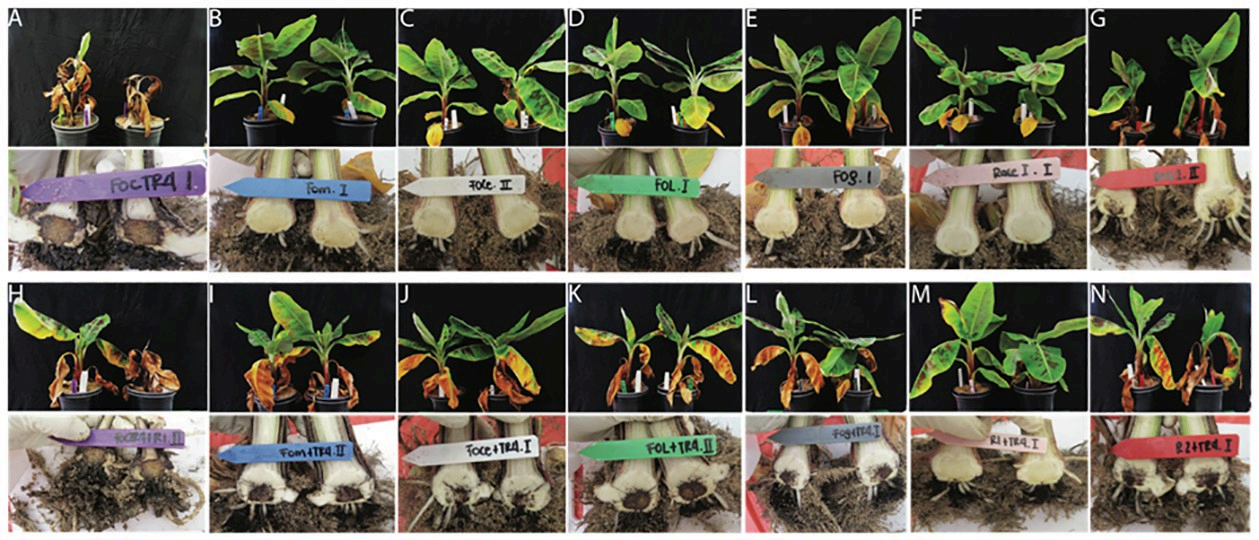
Disease development on Cavendish ‘Grand Naine’ with different *Fusarium* spp. (A-G): External and internal symptoms at six weeks after **single** inoculations with (A) *Fusarium odoratissimum* TR4, (B) *F*. *oxysporum* f. sp. *melongenae*, (C) *F*. *oxysporum* f. sp. *cepae*, (D) *F*. *oxysporum* f. sp. *lycopersici*, (E) *F*. *oxysporum* f. sp. *gladioli*, (F) *F*. *oxysporum* f. sp. *cubense* R1, and (G) *F*. *tardichlamydosporum* R2. (H-N): External and internal symptoms at six weeks after **primed** inoculations as follows (H) First inoculation with TR4 followed by challenging with R1 and (I-N) first inoculation as per (B-G) followed by a challenge with TR4.

We investigated the potential induction of resistance by different incompatible interactions with ‘Grand Naine’ as well as a series of interactions with non-pathogenic *Fusarium oxysporum* formae speciales on subsequent treatments with TR4 (Table 1). In initial infection trials, no infection symptoms were observed at 6 weeks after inoculation (wai) with the non-pathogenic strains or with the incompatible banana pathogenic strains (Table 1; Fig 1). However, severe symptoms were observed after inoculating with the compatible TR4 strain as well as with a R2 strain (Tables 1 and 2; Fig 1). Thus, all *Fo* strains were potentially suitable for the induction of hypothetical cross-protection except for the Race 2 strain as it resulted in FWB symptoms.

We then tested the cross-protection inducing capacity of these strains to TR4 by performing pre-inoculation treatments. Leaf chlorosis and wilting were observed in all treatments, at 3 wai except for the R1 pre-inoculated plants and the controls. At 6 wai, DIs of all plants exceeded 50%, with intense corm discoloration, severe chlorosis, wilting and other typical symptoms (Fig 1I–1N), with the exception of the R1 pre-treated plants which had lower DI values close to 20% (Tables 2 and 3; Fig 1M). The reverse treatment (first TR4 and then R1) did not result in reduced DIs. Plants pre-treated with R2 alone showed high DIs ranging from 60–75% (Fig 1G). Finally, all plants that developed symptoms were positive for TR4 molecular tests, but the corms from plants that were pre-treated with R1 and subsequently with TR4 tested negative.

**Table 3:** Effect of R1 and TR4 inoculations on the development of Cavendish ‘Grand Naine’.

Treatment	Chlorosis (%)	Score	DI^1^	Height (cm)	Dia^2^ (mm)	Foliage (cm^2^)	DW^3^
Control	27,82^a^	1^a^	00.1^a^	16.0^a^	24.29^c^	1592^c^	313.7^c^
R1	46.13^b^	2^b^	20.0^b^	21.83^b^	21.45^b^	1183^b^	257.3^b^
R1 + TR4	46.72^b^	2^b^	21.67^b^	23.60^c^	22.34^b^	1166^b^	286.7b^c^
TR4	92.06^c^	5.8^c^	96.65^c^	25.05^d^	15.08^a^	552^a^	138.3^a^

^1^DI = disease index.

^2^Dia = pseudostem diameter (mm).

^3^DW = dry weight (g).

**Fig 2:**
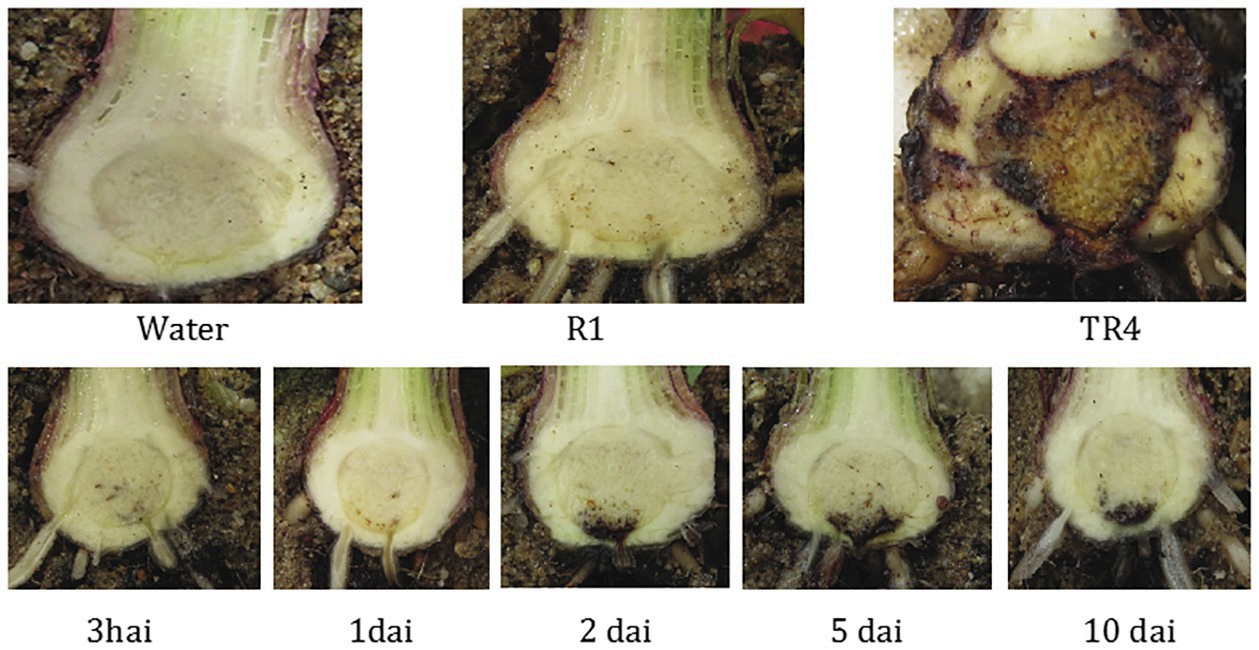
Durability of induced resistance in Cavendish banana cv. Grand Naine after pre-inoculating with R1. Cross sections of corms were conducted at six weeks after TR4 inoculation. Top row shows: water, R1 and *F*. *odoratissimum* TR4 inoculation controls. Bottom row shows: corms of R1 pre-inoculated plants that were challenged with TR4 after 3 hours, 1, 2, 5 and 10 days.

The experiment to test the duration of the induced resistance to TR4 by the R1 pre-treatment showed that extensive reduction of disease development was still observed when TR4 challenge inoculations were performed up to 10 days after R1 priming (Fig 2).

### Abiotic conditions do not affect the induced resistance by R1

**Fig 3:**
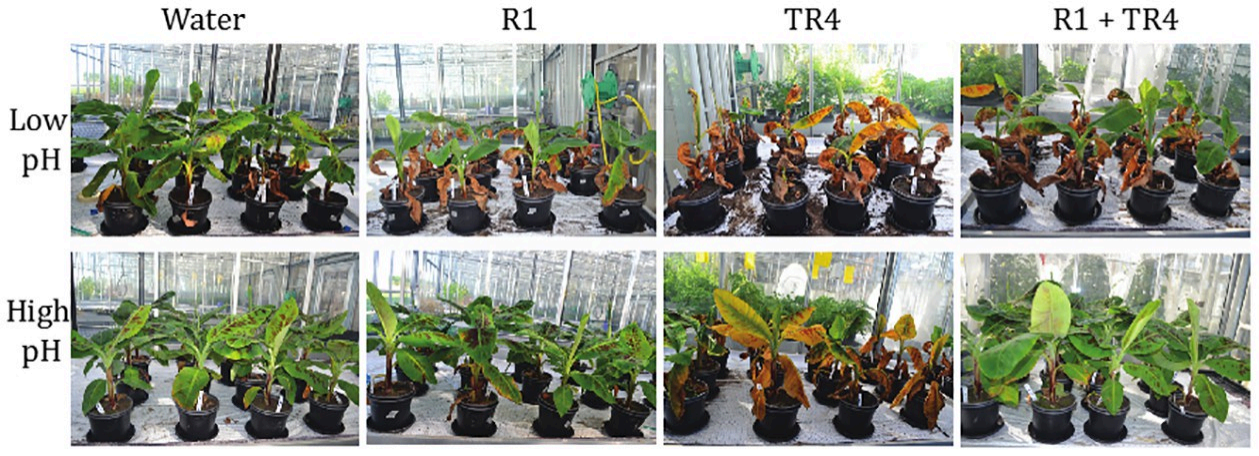
Fusarium wilt symptoms in ‘Grand Naine’ Cavendish banana six weeks after inoculation with R1. *F*. *odoratissimum* TR4 and a challenge inoculation (R1 + TR4) at pH 5 (top row) and pH 6 (bottom row).

We tested whether abiotic factors such as soil pH and nitrogen (N) levels influenced the significant reductions in disease severity caused by R1 pre-inoculations. Irrespective of the applied pH and N modulations, the induced resistance by R1 pre-inoculations reduced the disease severity caused by subsequent TR4 inoculations (Tables 4 and 5; Fig 3). At 6 wai, the DIs of R1 pre-inoculated plants had mean values close to 20 whereas those inoculated with TR4 alone had 96 (Table 3). At pH 6.0, the disease severity was very low in the R1 and R1 + TR4 treatment compared to the TR4 treatment (Table 4; S1 Table). At pH 5.2, disease symptoms were more developed in all treatments (including R1 and R1 +TR4) compared to high pH, but severity was still higher in the TR4 treatment when compared to R1 or R1+TR4 treatments (Fig 3; Table 4; S1 Table). Nutrition, however, did not affect the DIs, except for the R1 treatment where the DI was significantly lower under low nitrogen. Nitrogen fertilization affected plant growth as measured by plant height, but significant differences in stem diameter were only observed in the TR4 treatment (Table 5).

**Table 4:** Effect of soil acidity on FWB development in Cavendish ‘Grand Naine’, six weeks after inoculation with R1, TR4 and challenge inoculations (R1 + TR4).

Treatment	pH^1^	Chlorosis (%)	Score	DI^2^	Height (cm)	Dia^3^ (mm)	Foliage (cm^2^)	DW^4^
Water	6	20.32^a^	1^a^	00.1^a^	25.0^d^	1621^c^	337.0^de^	24.79^c^
	5.2	35.32^b^	1^a^	00.1^a^	25.0^d^	1563^c^	290.3^d^	23.80^c^
R1	6	32.32^ab^	1^a^	1.68^a^	25.5^d^	1921^d^	327.8^de^	24.61^c^
	5.2	59.53^c^	3^b^	41.66^b^	18.12^bc^	445^a^	186.7^bc^	18.29^b^
R1 + TR4	6	27.58^ab^	1^a^	8.34^a^	27.49^e^	2019^d^	373.0^e^	25.51^c^
	5.2	65.87^c^	2.5^b^	31.67^b^	19.71^c^	314^a^	200.4^c^	19.17^b^
TR4	6	89.68^d^	5.7^c^	98.31^c^	14.65^a^	819^b^	143.9^ab^	14.56^a^
	5.2	94.44^d^	5.9 ^c^	94.68^c^	17.35^b^	286^a^	132.7^a^	15.59^a^

^1^Low pH is ~5, high pH is ~ 6,

^2^DI = disease index,

^3^Dia = pseudostem diameter,

^4^DW = dry weight (g).

**Table 5:** The effect of different nitrogen (N) levels on FWB development in Cavendish ‘Grand Naine’ six weeks after inoculation with *Fusarium* R1, *F*. *odoratissimum* TR4 and challenge inoculations (R1 + TR4).

Treatment	N^1^	DI^2^	Height (cm)	Diameter (cm)
Water	Low	00.1^a^	22.95^de^	23.46^cd^
	Mid	00.1^a^	25.82^f^	24.96^d^
	High	00.1^a^	26.38^f^	24.47^d^
R1	Low	5.01^ab^	19.90^c^	21.64^c^
	Mid	30 ^d^	24.66^ef^	21.10^c^
	High	30^d^	20.9^cd^	21.60^c^
R1 + TR4	Low	25c^d^	21.50^cd^	21.64^c^
	Mid	20^cd^	24.19^ef^	31.86^c^
	High	15.0^bc^	25.11^ef^	23.56^cd^
TR4	Low	97.48^e^	17.0^b^	16.55^b^
	Mid	97.48^e^	16.50^ab^	15.90^d^
	High	97.48^e^	14.50^a^	12.78^a^

^1^Low, medium and high N levels are 0, 0.28 and 0.85 g/plant/week,

^2^DI–disease: plant height,

^3^Pseudomstem diameter.

### Analysis of the transcriptome of the interaction between R1 and TR4 on ‘Grand Naine’

We performed an RNAseq analysis with the aim of trying to understand the gene regulation during the initial stages after inoculations with individual TR4 and R1 strains as well as various challenge inoculation with both strains. We generated in total 317,020,712 RNAseq reads from three biological replicates of infected root tissues of the various treatments. Principal component analyses of regularized log-transformed TPM values revealed minimal variability between replicates of individual samples (S1 Fig). Differentially expressed genes were observed in comparison with the water controls at the different time points even upon TR4 inoculation (30 min after R1 pre-inoculation). In general, a higher number of transcripts was up- or down-regulated during the initial states of the infection for all the interactions, but numbers stabilized after one day. At time 0h (30 min after R1 Inoculation) 2162, 1754 and 2015 genes were differentially expressed in the plant when inoculated with Race 1, TR4 and the R1 + TR4 interaction, respectively. In total 207 genes were differentially up-regulated in plants inoculated with TR4, while only 79 were down-regulated. After R1 treatment, 99 genes were up-regulated, and 205 genes were down- regulated. The R1 + TR4 showed that in total 284 and 240 genes were up- and down-regulated, respectively (S2 Fig). In total 1,023 genes showed alteration in all three treatments, with 786 up and 237 down genes being commonly up- or down- regulated, respectively.

**Fig 4:**
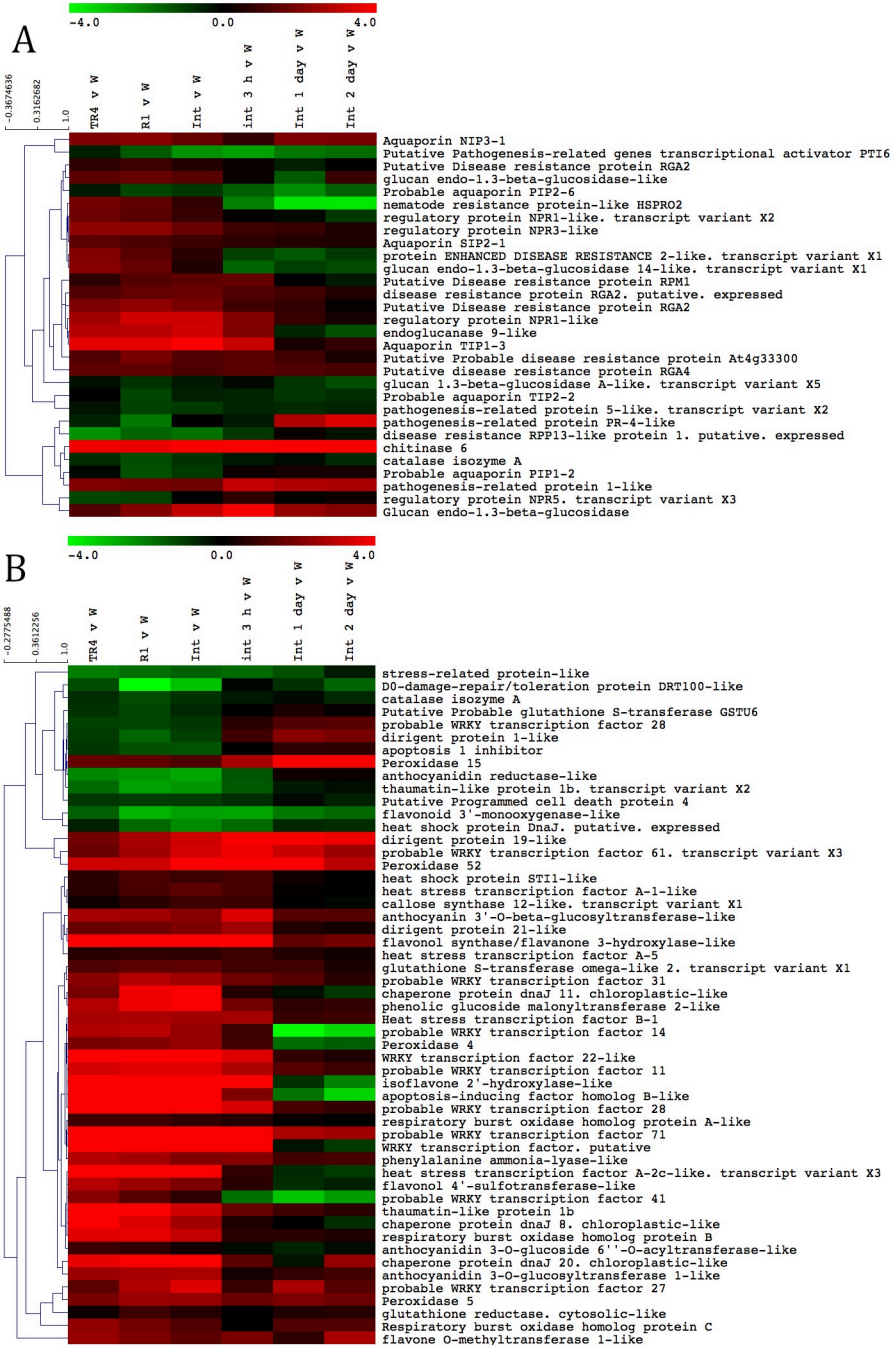
Transcriptional analyses of pathogenesis related genes in the interactions between Cavendish ‘Grand Naine’ and R1, *F*. *odoratissimum* TR4 and the challenge inoculation R1 + TR4. Hierarchical clustering analysis of representative potential pathogenesis related genes (Panel A) and hypersensitive response genes (Panel B). The heat plots represent significant (P < 0.05) average expression differences between the water control and TR4, R1 and R1 + TR4 at 0, 3 hours, 1 day and 2 days after inoculation. Bars represent upregulated genes (red) and downregulated genes (green).

To identify genes that are expressed upon the pre-inoculation, we focused on different genes, matching different functions, including pathogenesis related, hormone signalling and receptor-like genes. In general, most genes were similarly regulated for R1, TR4 and the R1 + TR4 interaction up to the first day, but thereafter the expression of the majority of genes was not different from the controls. Among the aforementioned groups of up-regulated genes, we identified genes encoding a thaumatin-like protein, a chitinase-6-like, an aquaporin, an endoglucanase-9 and β- 1–3 endoglucanase-like proteins as well as several peroxidase-like and phenylalanine ammonia lyase-like proteins (Fig 4). The chitinase-6 like and the β-1-3 endoglucanase like proteins were up-regulated across all treatments up to 2 dpi. These data suggest that defence responses were similarly induced in all treatments. Interestingly, two homologues of the resistance gene analogue *RGA2* and one homologue of *RGA4* genes were up-regulated in all treatments until 2 dpi.

**Fig 5:**
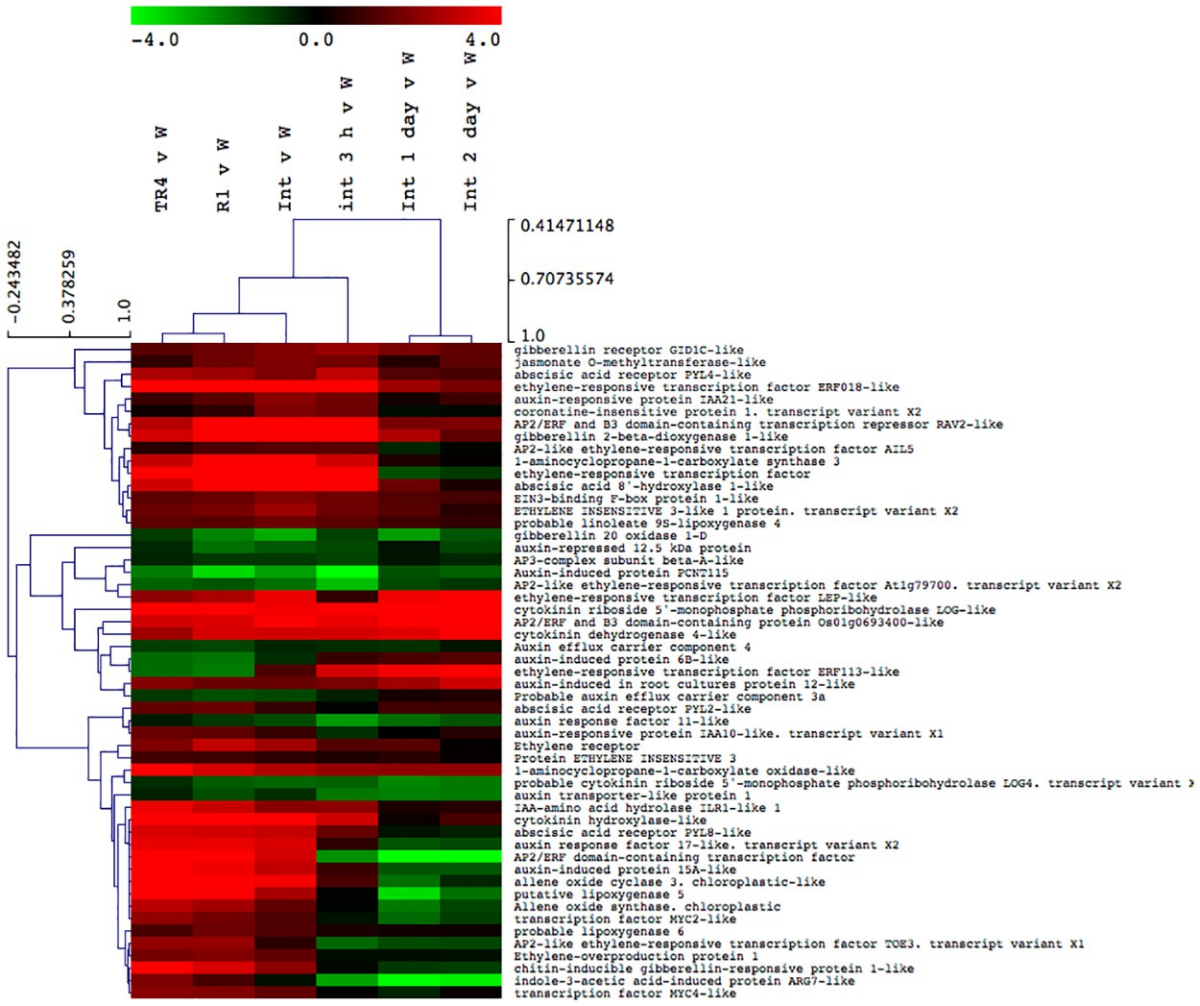
Transcriptional analyses of hormone response pathway related genes in the interactions between Cavendish ‘Grand Naine’ and R1, *F*. *odoratissimum* TR4 and the challenge inoculation R1 + TR4. Hierarchical clustering analysis of representative hormone response pathway. The heat plots represent significant (P < 0.05) average expression differences between the water control and TR4, R1 and R1 + TR4 at 0, 3 hours, 1 day and 2 days after inoculation. Bars represent upregulated genes (red) and downregulated genes (green).

We also searched for clues which plant signalling pathway could be involved in the induced resistance response. Until 3 dpi several hormone pathways were induced in the R1, TR4 and R1 + TR4 treatments, but the ethylene response pathway seemed to be most prominently up-regulated, involving several genes such as the *ERF018-like* ethylene-responsive transcription factor, the *AP2/ERF* transcription repressor as well as the *ACC synthase* gene. Interestingly, also several gibberellin syntheses-associated genes were strongly up-regulated including a *gibberellin 2-beta-dioxygenase 1-like* gene and the gibberellin receptor *GID1C like* (Fig 5). Finally, we identified up-regulation of a jasmonate receptor (Coronatine- insensitive protein) and a jasmonate O-methyltransferase-like protein in the jasmonate response pathway and *NPRace 1-like* regulatory protein as well as a *nudix hydrolase 8-like* gene in the salicylic acid pathway.

## Discussion

Contemporary banana production is threatened by the dissemination of *F*. *odoratissimum* TR4 that destroys Cavendish varieties as well as many local cultivars [58, 63]. As such, it is another demonstration of a new fungal species that causes great damage, similar to the recently described amphibian fungal pathogens [64]. Taking into account previous experiences with FWB [65], it is likely not an exaggeration to claim that TR4 threatens food security in many countries and has the potential to seriously affect global Cavendish production. Despite this experience, the overall response to TR4 is very similar to the previous strategies used to manage the FWB epidemic in ‘Gros Michel’ caused by R1 strains [21, 65].

As many agro-ecological approaches failed to show enduring efficacy, we focused in this study on the potential of induced resistance. In other crops, such as melon, tomato and chickpeas, ISR has shown potential to protect plants to different strains of *Fusarium* spp. causing wilts [35, 38, 39, 41, 66–68]. We hypothesized that utilizing the incompatible response of ‘Cavendish’ cultivars to R1 which has endured already for decades despite cultivation in highly infested soils [7], might be a potential way to reduce the impact of TR4 [55, 69–73].

As expected, we observed a compatible interaction of ‘Grand Nain’ cultivar with TR4, but unexpectedly there was a moderately susceptible response to R2 [24]. It is even more surprising as Moore *et al*. (1993) [27] considered this particular strain to be R1. Hence, it is not always clear which species actually causes damage in plantations. For instance, it is still unknown which *Fusarium* species was responsible for the epidemic in ‘Gros Michel’ [12, 65, 74]. The first original reports indicate that germplasm susceptible to R1 was resistant to R2 and *vice versa* (Stover 1962). Our R2 strain was isolated from symptomatic Bluggoe accessions (ABB) but has also been identified on Silk bananas (AAB) as well as ‘Pisang Nangka’ (AAA) and Inarnibal (AA) [7, 26, 75, 76]. Therefore, Ordóñez et al., (2018), considered that the current race nomenclature of banana infecting *Fusarium* spp. requires a thorough revision as multiple species can infect ‘Cavendish’ varieties under greenhouse conditions. Clearly, various genotype-environment interactions complicate race identification, which warrants greenhouse trials under repeatable and controlled conditions. It is well known that avirulent *Fusarium* genotypes can cause disease on Cavendish cultivars during particular abiotic conditions. For instance, Costa et al. (2015) isolated several normally avirulent strains from plants with FWB-like symptoms under severe drought. However, none of these isolates caused disease under optimal greenhouse conditions and they tested negative for TR4 with a molecular diagnostic.

We consistently showed that pre-inoculations with R1 significantly reduced the levels of infection caused by subsequent TR4 inoculations additionally Cavendish plants treated with R1 showed improved growth compared to controls (Water), seems like priming effect induces better performance of the plants suggesting that *Foc* R1 could act as a growth promotor. Moreover, the effect was maintained under different pH and nutrition levels and endured for at least 10 days. We, therefore, consider R1 as a potential inducer of systemic acquired resistance to TR4, which warrants further and more detailed analyses. Very similar observations have been reported in other systems involving viral diseases and other fungal diseases [77–81]. Cross protection against *F*. *oxysporum* has been extensively reported in several hosts, with different activators including *Verticillium* ssp., saprophytes or even other *Fo formae speciales*. Other studies have confirmed the reduction of disease severity caused by *Fusarium* after plants were pre-treated with a non-pathogenic organism [41, 66, 67]. Isolates of *F*. *oxysporum* have been investigated on cucumber, chickpea, and several other hosts affected by Fusarium wilts [38, 39, 41, 43, 66, 82–85]. In general, strains mediating induced resistance are usually closely related to the pathogenic strains [82, 83]. The most effective inducers of resistance often belong to the same fungal species or *forma specialis* [35, 66, 86]. For the *Fusarium*–banana pathosystem, Wu et al. (2013) observed that *in vitro* plants of the Cavendish ‘Brazil Xiangjiao’ were resistant to TR4 after challenging with R1. In our study, cross protection was only induced by R1 pre-treatments, as simultaneous inoculations of R1 and TR4 rapidly developed disease, similar to TR4 controls or to a TR4 inoculation followed by a treatment with R1. This suggests that protection does not result merely from antagonism or competition between the two strains.

Previously, three hypotheses were proposed as possible mechanisms of cross protection: competition for nutrients, competition for infection sites at the root surface and induction of a systemic host resistance response [43, 83, 87]. Our data suggest that in our system systemic induced resistance is the most likely explanation since no effect was observed neither with the other formae speciales nor with the biocontrol strains *Fo*47 (FOSC clade 3) and *Fo*618-12. Thus, despite the fact that these strains did not affect banana, except for small lesions incurred by *Fog*, they did not reduce the susceptibility to TR4. It is well known that various *Fusarium oxysporum* ff. spp. have a narrow host range and usually infect a single host [88–91]. Our trials have shown that the induced resistance to TR4 is exclusively related to R1 treatments, which apparently trigger the required resistance pathways upon inoculation. Moreover, none of the abiotic modulations affected the overarching induction of resistance by R1. Initially, R1 caused severe symptoms favored at low pH (also in the R1 + TR4 trial), but after some weeks plants recovered, and hence the symptoms were most likely resulting from infection attempts of R1 under abiotic stress, which is also observed under field conditions [30, 92, 93].

In our trials, low soil pH favoured the appearance of FWB symptoms in all treatments compared to high pH except for the controls, showing that pH is an important factor for disease development. This has been confirmed for banana [93] and other pathosystems in low soil pH [94–96] as well as at high soil pH [94–96]. As an example, Groenewald et al., (2006) showed that high soil pH reduced the incidence of FWB, while the source of nitrogen fertilizer also affected disease development. In general, pH is an important soil quality indicator in banana plantations and higher productions are obtained at higher soil pH [94]. We observed some effects of N levels, but only in the trials involving R1. This is in agreement with field data accumulated during the previous FWB epidemic in ‘Gros Michel’ [20, 95]. The augmented disease levels at pH 5.2 could be due to varying bacteria populations in soil, even under greenhouse conditions, or to nutritional effects in the rhizosphere such as a decrease of micronutrients that are essential for growth, sporulation and virulence of *Fusarium* pathogens [96–98].

Thus, we have shown that the resistance induction could theoretically be used for enhanced FWB management. However, in reality it is not possible to use the R1 strain in non-indigenous countries due to phytosanitary regulations. Moreover, upscaling our experiments to field applications are impossible. Hence, future studies should unveil the underlying molecular mechanism of cross protection in order to understand the phenomenon and to search for alternative ways to induce systemic resistance responses without explicitly using a pathogenic strain. Such a strategy would be one of the manifold contributing factors to disease management in Cavendish plantations affected by TR4. These should include trials to identify whether living inoculum is necessary for cross protection compared to the merely physical presence of R1 [99] and also address the effects resistance inducers have–such as jasmonate or salicylic acid as well as other pathogens and pests—as induced resistance is rather nonspecific [36, 48, 68, 77, 100, 101].

Finally, our transcriptome analyses are a first approach to understand the molecular mechanisms involved in the R1 induced cross protection to TR4. In general, the number of differentially expressed transcripts was in accord with similar studies in other pathosystems [102–105]. Defence related genes such as a chitinase, thaumatin, and peroxidases were clearly induced in the R1 and R1 + TR4 and TR4 treatments. This suggests that systemic induced resistance is the most probable mechanism involved in cross protection in the *Fusarium—*banana pathosystem and that the time of induction is critical for achieving protection since simultaneous R1 and TR4 treatments did not reduce disease development. Our data suggest that if defence responses are induced prior to TR4 inoculation the latter is unable to cause severe infection. In addition, the ethylene, jasmonic acid and gibberellin synthesis pathways might be crucial for protecting cv. Grand Naine plants from TR4 upon R1 induced resistance. This complies with the observations of Wu et al. (2013) in their *in vitro* pathosystem model where they identified systemic expression of PR genes regulated by these pathways, suggesting that several pathways could be involved in the resistance response. They observed that the total salicylic acid content in roots of banana plantlets increased after leaf inoculation with R1, suggesting cross talk between the different pathways.

Peraza-Echeverria et al. (2008) [106] assessed the expression of the resistance gene analogues (RGAs) by RT-PCR in banana leaf and root tissues. RGA1, 3 and 5 showed constitutive expression profiles in both resistant and susceptible plants to TR4, whereas no expression was detected for *RGA4*. In contrast, *RGA2* expression was exclusively observed in plants with resistance to TR4. *RGA2* is a non-TIR-NBS-LRR plant disease resistance protein that is involved in resistance to TR4 in several banana accessions, including *M*. *acuminata* spp. *malaccensis* [34]. In our experiments, we observed upregulation of *RGA2*-like genes in all treatments. Hence, *RGA2* could be a key factor involved in both R1 and TR4 resistance. The transfer of *RGA2* from *M*. *acuminata* spp. *malaccensis* to Cavendish has been proven to result in plants with resistance to TR4 both in greenhouse experiments as well as in extensive field trials [34].

To date, there are no commercial cultivars resistant to Fusarium wilt in banana TR4 with similar levels of resistance as Cavendish to Fusarium Race 1, hence the importance and benefit on studying the presence of fusarium wilt in banana, the favourable environmental conditions and the mechanism of the induced resistance among other factors (109). Our results could serve as the basis for future lines of research by molecular plant scientists in Colombia and Peru where Fusarium TR4 has been recently described [107, 108]. Such research would be an essential contribution in adjacent banana areas of Latin America [109] to avoid the spread and devastation of banana plantations.

Taken together, we have shown that R1 is an important inducer of resistance to TR4 in Cavendish ‘Grand Naine’ plants. However, R1 is still a major concern in many banana producing regions and hence not suitable as a biocontrol agent. Moreover, application of other R1 strains in our trials did not induce the same levels of resistance to TR4 (not shown). Hence, it seems that the utilized R1 strain from Brazil has this capacity, which warrants further investigation and characterization to understand the underlying mechanisms of induced resistance to TR4 in order to evaluate whether these can be used as a component of overall FWB management. Fundamental understanding of these mechanisms could also apply to other banana varieties affected by other FWB associated *Fusarium* spp., which are important for domestic markets.

## Supporting information

S1 Fig Principal component analyses of regularized log transformed TPM values of replicates of individual samples. (DOCX)

S2 Fig The number of up-regulated and down-regulated genes upon pre-inoculation Cavendish ‘Grand Naine’ with *F*. *oxysporum* f. sp. *cubense* R1, followed by challenging with *F*. *odoratissimum* TR4 after 30 min. The Venn diagram shows the total number of differentially expressed genes as well as the genes that are similar between the various treatments, compared to the water control, immediately after TR4 challenge inoculations (Time 0 hai). Red arrows and green arrows indicate upregulated and downregulated gene numbers, respectively.
(DOCX)

S1 Table The effect of soil acidity and nitrogen (N) levels on FWB development in Cavendish ‘Grand Naine’ six weeks after inoculation with *Fusarium* R1, *F*. *odoratissimum* TR4 and challenge inoculation (R1 + TR4). (DOCX)
