# Supplementary material for: Induced resistance to Fusarium wilt of banana caused by Tropical Race 4 in Cavendish cv Grand Naine bananas after challenging with avirulent Fusarium spp
Source: PLoS One. 2022 Sep 21;17(9):e0273335. doi: 10.1371/journal.pone.0273335 (PMC9491598; doi:10.1371/journal.pone.0273335)
Supplement: S1 Fig — (DOCX) [file pone.0273335.s001.docx]

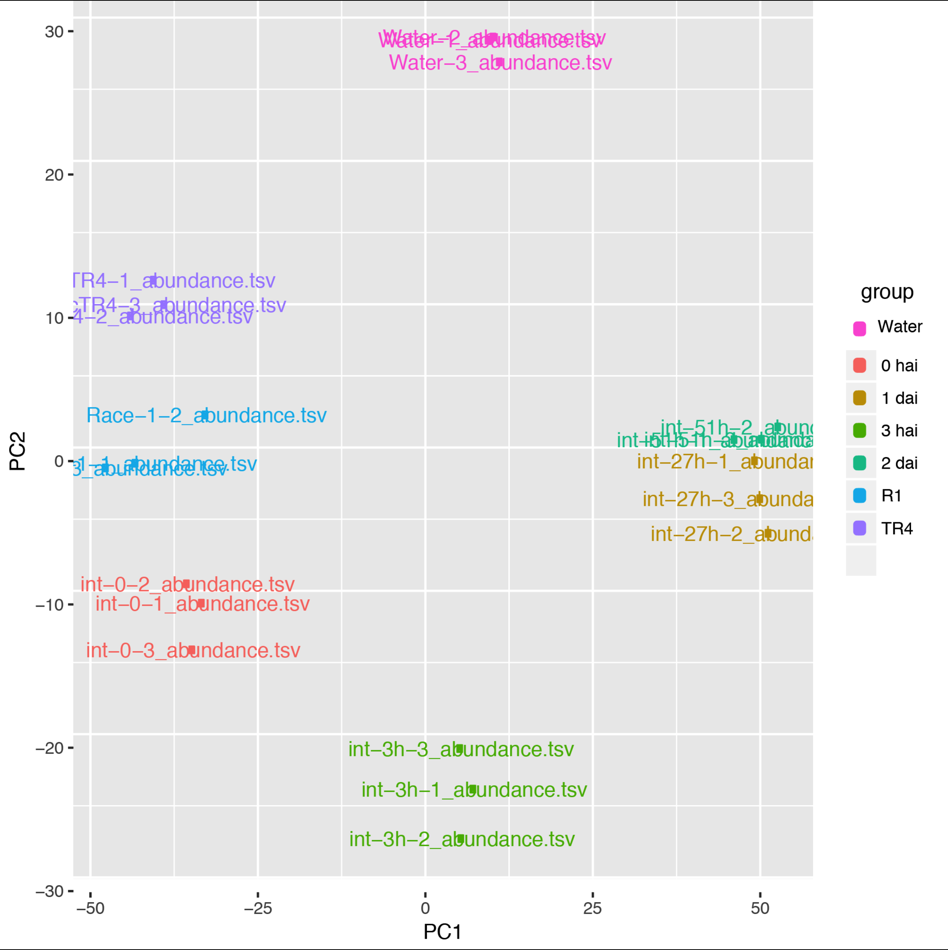


**S1 Figure.** Principal component analyses of regularized log transformed TPM values of replicates of individual samples.
