# Supplementary material for: Induced resistance to Fusarium wilt of banana caused by Tropical Race 4 in Cavendish cv Grand Naine bananas after challenging with avirulent Fusarium spp
Source: PLoS One. 2022 Sep 21;17(9):e0273335. doi: 10.1371/journal.pone.0273335 (PMC9491598; doi:10.1371/journal.pone.0273335)
Supplement: S2 Fig — The Venn diagram shows the total number of differentially expressed genes as well as the genes that are similar between the various treatments, compared to the water control, immediately after TR4 challenge inoculations (Time 0 hai). Red arrows and green arrows indicate upregulated and downregulated gene numbers, respectively. (DOCX) [file pone.0273335.s002.docx]

**S2 Figure.** The number of up-regulated and down-regulated genes upon pre-inoculation Cavendish ‘Grand Naine’ with *F. oxysporum* f. sp. *cubense* R1, followed by challenging with *F. odoratissimum* TR4 after 30 min. The Venn diagram shows the total number of differentially expressed genes as well as the genes that are similar between the various treatments, compared to the water control, immediately after TR4 challenge inoculations (Time 0 hai). Red arrows and green arrows indicate upregulated and downregulated gene numbers, respectively.
