# Supplementary material for: Induced resistance to Fusarium wilt of banana caused by Tropical Race 4 in Cavendish cv Grand Naine bananas after challenging with avirulent Fusarium spp
Source: PLoS One. 2022 Sep 21;17(9):e0273335. doi: 10.1371/journal.pone.0273335 (PMC9491598; doi:10.1371/journal.pone.0273335)
Supplement: S1 Table — (DOCX) [file pone.0273335.s003.docx]

**S1 Table. The effect of soil acidity and nitrogen (N) levels on FWB development in Cavendish ‘Grand Naine’ six weeks after inoculation with *Fusarium* R1, *F. odoratissimum* TR4 and challenge inoculation (R1 + TR4).**

| **Treatment** | **pH** | **N** | **Chlorosis**  **(%)** | **DI^1^** | **Height**  **(cm)** | **Diameter (cm)** | **Foliage**  **(cm^2^)** | **Biomass**  **(g)** |
| --- | --- | --- | --- | --- | --- | --- | --- | --- |
| Water | 5.2 | Low | 25 | 0 | 23.3 | 24.0 | 1422.6 | 293.8 |
|  |  | Mid | 27 | 0 | 25.8 | 23.8 | 1259.0 | 291.2 |
|  |  | High | 52 | 0 | 25.8 | 23.7 | 1574.0 | 291.6 |
|  | 6 | Low | 28 | 0 | 22.3 | 23.0 | 1523.8 | 311.7 |
|  |  | Mid | 21 | 0 | 25.9 | 26.1 | 1878.0 | 364.3 |
|  |  | High | 12 | 0 | 27.0 | 25.2 | 1460.4 | 335.2 |
| Race 1 | 5.2 | Low | 39 | 10 | 17.8 | 18.7 | 414.4 | 189.7 |
|  |  | Mid | 68 | 55 | 20.7 | 18.1 | 336.4 | 211.3 |
|  |  | High | 73 | 60 | 15.9 | 18.0 | 281.2 | 159.2 |
|  | 6 | Low | 21 | 0 | 22.0 | 24.6 | 1937.5 | 344.4 |
|  |  | Mid | 30 | 5 | 28.6 | 24.1 | 1950.4 | 333.0 |
|  |  | High | 45 | 0 | 26.0 | 25.2 | 1875.0 | 306.1 |
| Race 1 + TR 4 | 5.2 | Low | 61 | 30 | 20.1 | 19.6 | 278.9 | 193.5 |
|  |  | Mid | 75 | 40 | 19.1 | 18.0 | 253.6 | 195.9 |
|  |  | High | 62 | 25 | 19.9 | 20.0 | 346.6 | 211.7 |
|  | 6 | Low | 19 | 20 | 22.9 | 23.7 | 1868.5 | 384.7 |
|  |  | Mid | 31 | 0 | 29.3 | 25.7 | 2111.6 | 389.4 |
|  |  | High | 33 | 5 | 30.4 | 27.1 | 2075.4 | 344.8 |
| TR4 | 5.2 | Low | 100 | 100 |  | 16.9 | 77.8 | 102.7 |
|  |  | Mid | 88 | 90 | 14.0 | 13.9 | 117.4 | 167.6 |
|  |  | High | 96 | 95 | 18.0 | 15.9 | 148.1 | 127.6 |
|  | 6 | Low | 84 | 95 | 14.0 | 15.7 | 726.6 | 151.1 |
|  |  | Mid | 96 | 100 | 17.5 | 16.9 | 1123.1 | 177.1 |
|  |  | High | 89 | 100 | 11.0 | 11.0 | 442.8 | 103.5 |

^1^DI = disease index
